# The Spiked Helmet Sign Predicting a Poor Outcome in a Patient with Non-Myocardial Infarction ST-Segment Elevation

**DOI:** 10.3390/medicina57111184

**Published:** 2021-11-01

**Authors:** Shu-Yu Shih, Yueh-Tseng Hou, Po-Chen Lin, Yu-Long Chen, Da-Sen Chien, Giou-Teng Yiang, Meng-Yu Wu

**Affiliations:** 1 Department of Emergency Medicine, Taipei Tzu Chi Hospital, Buddhist Tzu Chi Medical Foundation, New Taipei 231, Taiwan; 99311150@gms.tcu.edu.tw (S.-Y.S.); brianann75@gmail.com (Y.-T.H.); taipeitzuchier@gmail.com (P.-C.L.); yulong0129@gmail.com (Y.-L.C.); sam.jan1978@msa.hinet.net (D.-S.C.); gtyiang@gmail.com (G.-T.Y.); 2 Department of Emergency Medicine, School of Medicine, Tzu Chi University, Hualien 970, Taiwan

**Keywords:** spiked helmet sign, ST-segment elevation myocardial infarction, subarachnoid hemorrhage, intraabdominal bleeding

## Abstract

Spiked helmet sign is a novel electrocardiogram marker that reflects a poor prognosis, and may mimic myocardial infarction, especially in patients with an acute alteration of mental status or out-of-hospital cardiac arrest. In cases where a spiked helmet sign is missed, there may be a delay in surgical intervention for the underlying conditions because of unnecessary cardiac catheterization. In addition, antiplatelet agents for acute coronary syndrome in such cases can lead to catastrophic complications. Therefore, early recognition of spiked helmet sign is useful for timely correction of the underlying disease and prevention of poor outcomes. Herein, we describe a rare case of a patient with internal bleeding and subarachnoid hemorrhage presenting with spiked helmet sign on an electrocardiogram.

## 1. Introduction

An acute ST-elevation (STE) myocardial infarction is a common finding in acute coronary syndrome, and it suggests occlusion of coronary arteries, which causes transmural myocardial ischemia. However, STE may also be present in critically ill patients. In 2011, Littmann et al. [1] reported eight cases with a dome-and-spike pattern ST-segment elevation. The appearance was similar to that of the Pickelhaube, the German military spiked helmet. A detailed pathogenesis of the association between spiked helmet sign and poor clinical outcomes is still uncertain. According to a few case analyses, a pulsatile diaphragmatic motion induced by the inferior wall of the left ventricle or triggering the left phrenic nerve may cause ST segment alteration, which is commonly noted in the inferior leads (II, III, and aVF). Spiked helmet sign commonly occurs in critical noncardiac illnesses and can be seen in the inferior leads (II, III, a nd aVF). Cardiac serum markers can be used to rule out type I myocardial infarction in patients whose electrocardiograms (ECGs) show spiked helmet sign. In clinical outcome analyses, spiked helmet sign is a novel ECG marker that indicates a poor prognosis and is associated with a high mortality rate of up to 75% [1]. Therefore, early recognition of spiked helmet sign is useful for timely correction of the underlying disease and the prevention of poor outcomes. Herein, we describe a rare case of a patient with internal bleeding and subarachnoid hemorrhage (SAH) presenting with a spiked helmet sign on his ECG. The etiology and ECG features of the spiked helmet sign are discussed.

## 2. Case Presentation

**Figure 1:**
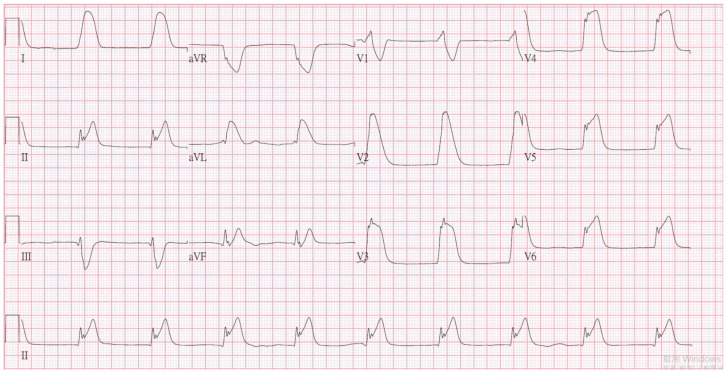
The initial electrocardiogram revealed ST-segment elevation in leads II and V_3-6_, and there was a spiked helmet sign.

**Figure 2:**
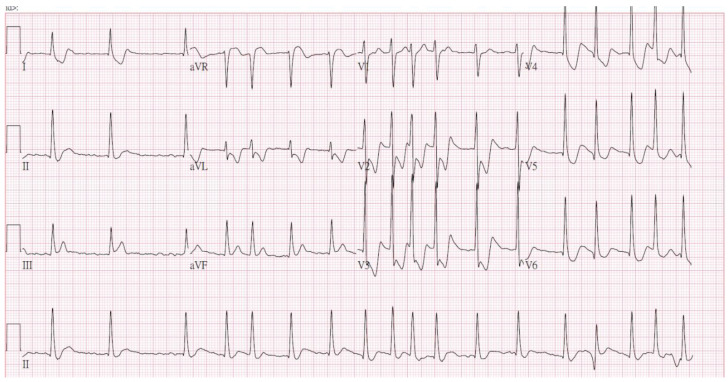
The follow-up electrocardiogram showed atrial fibrillation with a rapid ventricular response and ST-segment depression in V_2-6_.

**Figure 3:**
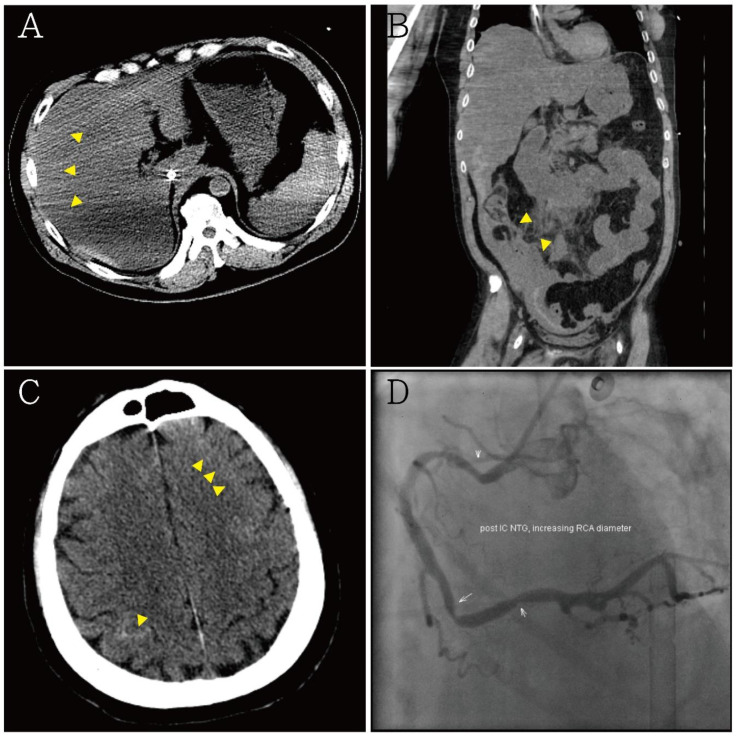
(**A**,**B**) Abdominal computed tomography showing hemoperitoneum due to internal bleeding (yellow arrowheads), liver laceration was suspected; (**C**) brain computed tomography showing minimal bilateral sulci subarachnoid hemorrhage (yellow arrowheads); (**D**) diagnostic coronary angiography showing only coronary artery spasm and native left and right coronary artery diameters with an increased myocardial bridge after intra-coronary nitroglycerin injection. There was no occluded segment or flow-limiting lesion.

A 54-year-old man presented with loss of consciousness and persistent bradycardia after a traffic accident. He had a history of hypertension and was on regular medications. He had no history of cardiovascular disease at a personal and family level. He was observed to have facial abrasions and epistaxis. He had no cerebrospinal fluid otorrhea or rhinorrhea, and no deformity of the extremities. After being admitted to the emergency department, he was noted to have bradycardia (40–46/min), and his blood pressure could not be detected. After four minutes, the cardiac monitor showed pulseless electrical activity. Timely cardiopulmonary resuscitation was performed, and the patient returned to spontaneous circulation. On neurological examination, his Glasgow Coma Scale score was E_1_V_T_M_1,_ and he had a 3.5 mm bilateral pupil size without light reflex. An electrocardiogram (ECG) revealed ST-segment elevation in leads II and V_3-6_, with a spiked helmet sign (Figure 1). Acute myocardial infarction was suspected. Bedside cardiac ultrasound revealed grossly normal wall motion of the left ventricle under inotropic agents. The interventricular septum and left ventricular posterior wall thickness were noted, and there was no pericardial effusion. A follow-up ECG revealed atrial fibrillation with a rapid ventricular response and ST-segment depression in V_2-6_ (Figure 2). Cardiac enzyme studies revealed no elevation of the level of the highly sensitive troponin I (5.5 pg/mL (normal range <17.5 pg/mL). Abdominal computed tomography revealed hemoperitoneum due to internal bleeding (Figure 3A,B). Liver laceration was suspected. Brain computed tomography revealed mild cortical brain atrophy and minimal bilateral sulci subarachnoid hemorrhage (Figure 3C). The patient was promptly placed on extracorporeal membrane oxygenation (ECMO) due to a poor response to inotropic agents. Diagnostic coronary angiography revealed only coronary artery spasm and a myocardial bridge of the left anterior descending artery (Figure 3D). There were no occluded segments or flow-limiting lesions. After the administration of an intracoronary nitroglycerin injection, the native left coronary artery and right coronary artery diameters significantly increased. A left ventriculogram showed mild mitral valve regurgitation and mild hypokinesia of the inferior wall. The left ventricular ejection fraction was 60% under ECMO and inotropic agents. However, even with adequate resuscitation, the patient remained with a low cardiac output and progressively went into shock; he eventually died.

## 3. Discussion

Cardiac ischemia causes metabolic dysfunction by blocking oxidative metabolism, which leads to the depletion of ATP and accumulation of lactic acid [2]. Under aerobic conditions, the accumulation of external K^+^ due to ATP-sensitive K channels (I_K-ATP_) triggers the cell to depolarize and shorten the action potential, prolong the effective refractory period, and decrease the conduction velocity [3]. Intracellular and extracellular acidosis, resulting from CO_2_ retention, lead to the activation of the Na^+^/H^+^ exchanger, resulting in over entry of Na^+^, and causing calcium overload by activating the Na^+^/Ca^++^ exchanger [4]. Most channel proteins, such as enzymes, may be impaired under acidic conditions. A consequent electrophysiological change from a decreased slow inward calcium current and activation of I_K-ATP_ presents with disparate responses in action potentials as currents flow across the boundaries of the ischemic myocardium. In an ECG recorded with a direct-current amplifier for acute coronary syndrome, the ST segment presented with elevation because of the shorter action potential in the ischemic part of the heart [5]. However, ECG findings of ST-segment elevation, such as spiked helmet sign, could be caused by various underlying pathologies, some of which may not be associated with cardiac ischemia. Initially, spiked helmet sign was described as an artifact that is commonly seen in the inferior leads (II, III, aVF) due to voltage alternation from pulsatile diaphragmatic motion induced by mechanical epidermal stretch from secondary etiologies [1]. Subsequent reports have also demonstrated its presentation in other chest leads [6,7]. It has also been hypothesized that the pathogenesis of spiked helmet sign involves acute increases in intraabdominal or intrathoracic pressure. Another hypothesis of spiked helmet sign, i.e., sympathetic overstimulation, has been suggested with other etiologies, such as sepsis and metabolic derangement. In a report by Aliyev et al. [8], a spiked helmet sign was seen on the ECG of a patient after left percutaneous stellate ganglion ablation. The authors assumed that the spiked helmet sign could have been induced by central sympathetic activation, which is similar to what happens in Takotsubo cardiomyopathy. Another study also suggested a potential relationship between Takotsubo cardiomyopathy and a spiked helmet sign [9]. Agarwal et al. reported another case of a non-cardiac illness with a spiked helmet sign [10]. The patient was diagnosed with acute respiratory distress syndrome; he was placed on mechanical ventilation, and an ECG showed the spiked helmet sign, which was due to the high positive end-expiratory pressure (PEEP) of up to 13 mmHg. A bedside echocardiography showed a preserved left ventricular ejection fraction without wall motion abnormalities. An acute rise in intrathoracic pressure with an increase in PEEP may also trigger a spiked helmet sign. In an article by Oluyadi et al. [11], a patient diagnosed with out-of-hospital cardiac arrest presented with spiked helmet sign after return of spontaneous circulation. A bedside echocardiogram showed no wall motion abnormalities. Spiked helmet sign seen in critically ill patients often indicates a very poor prognosis.

Acute central nervous system events may induce spiked helmet sign, which mimics acute coronary syndrome, especially in SAH [12]. In these conditions, it is difficult to make a distinction basing on clinical symptoms. Patients may present with acute alteration of mental status or out-of-hospital cardiac arrest. Missing the spiked helmet sign may delay surgical intervention because of unnecessary cardiac catheterization. In addition, antiplatelet agents for acute coronary syndrome can lead to catastrophic complications in such cases. Our patient had an uncommon electrocardiogram finding (a spiked helmet sign), intra-abdominal bleeding, and SAH. The ECG revealed ST-segment elevation in leads II and V_3-6_, as well as a spiked helmet sign. Due to traumatic out-of-hospital cardiac arrest, a whole-body CT scan revealed hemoperitoneum and SAH. Both etiologies could lead to a spiked helmet sign by the activation of pathologically pulsatile diaphragmatic motion and sympathetic overstimulation. Cardiac events in our patient were ruled out by cardiac serum markers and bedside ultrasound. Diagnostic coronary angiography confirmed these results. In addition, the prognosis of our patient was consistent with the previous results that reported that spiked helmet sign reflects poor outcomes. Therefore, we present this case to highlight a novel ECG marker. In patients with a spiked helmet sign, early identification of the etiologies, such as acute thoracic lesions, acute abdominal diseases, central nervous system events, QT prolongation, or sympathetic overstimulation, prevents delaying of the much-needed therapeutic strategies.

## 4. Conclusions 

Spiked helmet sign commonly occurs in critical noncardiac illnesses. In patients with a spiked helmet sign, early identification of the etiologies and carefully differentiation from acute coronary syndrome prevents delaying of the much-needed therapeutic strategies.

## Data Availability

Not applicable.
